# Large reductions in cesarean delivery rates in China: a qualitative study on delivery decision-making in the era of the two-child policy

**DOI:** 10.1186/s12884-017-1597-9

**Published:** 2017-12-04

**Authors:** Eileen Wang, Therese Hesketh

**Affiliations:** 1 Institute for Global Health, School of Public Health, Zhejiang University, 866 Yuhangtang Lu, Hangzhou, 310058 China; 2 Institute for Global Health, University College London, 30 Guilford St., London, UK

**Keywords:** Cesarean delivery, Two-child policy, Delivery decision-making, China

## Abstract

**Background:**

In 2010, China’s cesarean delivery (CD) rates increased to one of the highest in the world, a significant proportion of which were without medical indication. However, recent studies have indicated some declines, coinciding with national and local efforts to promote vaginal birth, as well as the relaxation of the one-child policy. Considering these trends, we aimed to qualitatively explore attitudes towards childbirth and experiences of delivery decision-making among women and physicians.

**Methods:**

Semi-structured interviews were conducted with 45 postpartum women and 7 healthcare providers at one county-level and one provincial-level maternity hospital in Zhejiang Province. We also collected routine data from 2007 to 2016 and observed doctor-patient interactions and hospital facilities as context for the interviews. Interviews were recorded, translated and transcribed into English, and then analyzed using a framework approach.

**Results:**

From 2007 to 2016, cesarean delivery rates at the county-level and provincial-level hospital decreased from 46% to 32% and 68% to 44%, respectively. For low-risk women, vaginal birth was the primary choice of delivery method, encouraged by doctors and nurse-midwives. Elective CD was not as widely accepted, in contrast to previous years. Women were aware of and took into consideration the consequences of CD for future pregnancies. Among those who delivered vaginally, women viewed the existing pain relief methods, epidurals and transcutaneous electrical nerve stimulation, with caution or uncertainty. Even when requested, epidurals were only given under certain circumstances. For multiparas with previous CD, repeat CD remains the norm. Both women and professionals were cautious about vaginal birth after cesarean delivery (VBAC) given the associated risks.

**Conclusion:**

In China, changes in family planning policy and efforts to promote vaginal birth have greatly changed the culture of delivery decision-making, leading to decreased CD rates. This demonstrates the powerful role social factors and public policy can play, and provides a model for other countries with high CD rates. Further research should explore changes in other reproductive decisions during this new multiparous era, particularly across provinces.

**Electronic supplementary material:**

The online version of this article (10.1186/s12884-017-1597-9) contains supplementary material, which is available to authorized users.

## Background

In 1993 the overall rate of cesarean delivery (CD) in China was 5%. By 2010 it had increased to national estimates of 40 to 50%, and rates as high as 70% in some urban areas [1–3]. This steep increase was driven by a host of structural and provider factors, including rapid economic development and urbanization, increasing hospitalization of births, large volume of care with limited resources, and financial incentives to perform CD [1, 4–7]. High rates of CDs without medical indication have also been driven by “social factors” or cesarean deliveries on maternal request (CDMR), which comprised 10% to 28% of all CDs in 2011 [5, 8, 9]. Studies demonstrated that women’s reasons for choosing cesarean included anxiety about labor, fear of pain, choice of an auspicious delivery date, and demand for a “perfect” child or birth outcome [4, 10, 11]. Still, there has been increasing concern about the rise in these unnecessary cesarean deliveries. In cases where CDs are non-medically indicated, maternal morbidity and mortality increases compared to cases of women delivering vaginally [2]. Furthermore, a CD for primiparous women increases the risk of uterine rupture, spontaneous miscarriage, abnormal placentation, and other complications in subsequent pregnancies [12].

Given these concerns, China began national and local public health efforts to decrease the CD rate through health promotion, practitioner training and tightening of hospital regulations [13–15]. More recent statistics have shown CD rates in some areas, particularly cities, have since declined by as much as 30% between 2008 and 2014 [16]. This decrease also coincided with the gradual relaxation of the one-child policy, which had been implemented in 1979, and restricted many families to one child [17]. In 2007, all provinces began to permit couples who were both only children to have two children, and in 2013, if one of the partners was an only child. From January 2016 all couples have been allowed two children [18]. The implications of the new policy include increased burden on maternity services, increases in deliveries to older women and women with previous CDs, and changes in reproductive counseling and decision-making [19].

While studies have documented these changes in CD rates, none have explored the changes in delivery decision-making in the context of both the two-child policy and efforts to limit unnecessary CDs. Therefore, the aim of this study was to understand how decisions about delivery method are made, from the perspectives of women and their healthcare providers. The specific objective was to explore attitudes of women and practitioners towards vaginal birth versus cesarean birth, including vaginal birth after cesarean (VBAC), in the era of the two-child policy. We additionally intended to explore women’s attitudes towards pain relief during labor and the role it plays in delivery decision-making, particularly as studies have shown how the fear of pain has been a significant contributor to CDMR [5, 11, 20]. We wished to understand changes in CD decision-making in conjunction with the childbirth experience, of which the relief of pain is an integral component.

## Methods

### Study sample

We conducted the study from November 2016 to April 2017 in Zhejiang, a relatively wealthy coastal province in Eastern China. Study sites were a provincial-level teaching hospital in Hangzhou, and a county-level hospital in Jiangshan. In China, hospitals are classified according to a three-tier system: a primary hospital is usually located in a township and contains less than 100 beds; a secondary hospital contains between 100 and 500 beds; and a tertiary hospital provides the most comprehensive healthcare services with a bed capacity of 500 or more [21]. In this case, the Hangzhou hospital is tertiary-level and a referral unit for high-risk pregnancies, serving a mainly urban population; the Jiangshan hospital is a secondary-level center, serving a mainly rural population. We chose the former because it has been shown in previous studies that tertiary-level hospitals serving an urban population were more likely to have had high rates of CDs and subsequent declines in recent years [3, 16]. We also selected a smaller hospital with a rural patient population to provide comparison and assess differences in rural versus urban attitudes.

### Study design

We conducted in-depth, semi-structured interviews with 45 postpartum women, 24 from Hangzhou and 21 from Jiangshan, which allowed us to reach saturation of themes. Nurses and physicians non-randomly identified women who were two to five days postpartum and who were of different parities and birth outcomes. Interviews were conducted on the wards, sometimes with family members present, using a previously-developed interview guide that included questions about the woman’s birth experience and views on vaginal versus cesarean birth. We also interviewed four nurse-midwives and three obstetricians about their experience working as health practitioners, and their views of the delivery decision-making process, particularly over time. Interview guides developed and used for this study can be viewed as a Additional files 1 and 2. Prior to each interview, we explained the purpose of the study and obtained participants’ verbal consent to be interviewed and recorded.

In addition to interviews, the researcher (EW) spent a month at each hospital observing and taking fieldnotes on doctor-patient relationships and the general environment of maternity care. During that time, the researcher informally interviewed health practitioners and key informants, observed outpatient clinics, inpatient wards and labor and delivery rooms, and attended prenatal classes. Hospitals’ routine data from 2007 to 2016, including numbers of births, cesarean deliveries, cesarean indication, maternal age and, if available, parity, were also collected. This provided context for participants’ responses.

### Analysis

Audio recordings of the interviews were translated and transcribed directly into English by the bilingual researcher (EW). We used previously-identified conceptual codes, guided by preset interview topics, such as their present and past childbirth experiences as well as attitudes towards delivery method, to analyze the data. Responses were extracted, sorted by parity and birth outcome, and then analyzed for patterns. Findings were summarized and organized into a larger explanatory framework based on similarities and differences in responses depending on parity, previous delivery method or hospital. These were compared to and integrated with fieldnotes. The study was approved by the Ethics Committee of Zhejiang University.

## Results

### Hospital settings

The Jiangshan maternity hospital has one obstetric ward with 72 beds, two operating rooms and one labor-delivery room. There are 15 obstetricians and 26 nurses across all maternity services. The number of births increased from around 2000 in 2007 to 2531 and 2703 in 2012 and 2016, respectively (Table 1). The CD rate was around 46% in 2007, which decreased to 32% in 2016. The percentage of repeat CD out of all CDs increased from 32% to 49% from 2012 to 2016 (data from before 2012 were not obtained). Although daytime epidural services have been available since 2011, only 7% of women delivering vaginally use them. Around 43% instead choose to use transcutaneous electrical nerve stimulation (TENS) on Traditional Chinese Medicine acupoints, available since 2015. Instrumental deliveries account for less than 1% of all vaginal births. Episiotomy rates decreased from 70% in 2007 to 30% in 2016. Women generally enter the labor-delivery room at around 3 cm dilated; family members are not allowed. VBAC is generally not allowed because of the lack of resources in case of emergency surgery.

**Table 1:** Collected Hospital Data, 2007–2016

Jiangshan Maternity Hospital^a^										
	2007	2008	2009	2010	2011	2012	2013	2014	2015	2016
Number of births						2531	2372	2649	2116	2703
Cesarean deliveries (% of births)						40%	40%	39%	37%	32%
Age > 35 years (% of births)						16%	20%	15%	11%	22%
Repeat CD (% of all CDs)						32%	35%	32%	40%	49%
Pain relief^b^ (% of all vaginal deliveries)						3%	15%	15%	29%	50%
Zhejiang Women’s Hospital										
	2007	2008	2009	2010	2011	2012	2013	2014	2015	2016
Number of births	10,106	10,271	10,961	11,414	12,090	14,801	14,332	18,115	14,598	20,534
Cesarean deliveries (% of births)	68%	67%	60%	54%	50%	51%	51%	46%	46%	44%
Age > 35 years (% of births)	7%	7%	8%	8%	8%	7%	10%	10%	16%	18%
Repeat CD (% of all CDs)	6%	7%	10%	12%	15%	18%	21%	30%	43%	51%
Epidurals^b^ (% of all vaginal deliveries)						39%	39%	31%	30%	26%
VBAC (% of eligible CDs)						2%	1%	1%	3%	5%

^a^Data from before 2012 from Jiangshan Maternity Hospital were unable to be collected. The approximate number of births and percentage of cesarean deliveries before 2012 were revealed anecdotally in interviews

^b^The pain relief data marked for Jiangshan includes the Traditional Chinese Medicine transcutaneous electrical nerve stimulation (TENS) after its implementation in 2015. Daytime epidural services have been available since 2011. For Zhejiang Women’s Hospital, only consecutive data for epidurals were reported, although they also offer other methods of pain relief and social support, such as doulas. Data from before 2012 were unavailable

The Hangzhou maternity hospital has five obstetric wards with a total of 500 beds. There are two large labor rooms, each with ten beds, and six delivery rooms, each with two beds, as well as 40 obstetricians and 60 nurse-midwives. The number of births increased from 10,106 in 2007 to 14,801 and 20,534 in 2012 and 2016, respectively (Table 1). The CD rate was 68% in 2007, and 44% in 2016. The percentage of repeat CD out of all CDs increased from 6% to 51% from 2007 to 2016. Around 40% of women use doula services. In 2016 26% of women delivering vaginally used epidurals, and 8% TENS, available since 2009. Around 5% of eligible women undergo VBAC, but only with an epidural and doula monitoring. In 2016, instrumental delivery rates were around 6%; episiotomy rates were 15%, down from 65% in 2012. Women enter the labor ward from the patient wards at around 3 cm dilated and are then transferred to the delivery room for second stage. Family members are sometimes allowed into the labor ward, depending on volume and time of day. Women are encouraged to move around and choose their position while laboring, but not for delivery. In addition to the wards, there are 20 “VIP,” private labor and delivery rooms for women and their families, which cost an average of 6300 RMB (1000 USD) per night.

### Interviews

Of the 45 postpartum women interviewed, 16 were primiparas, 29 multiparas, 24 delivered by cesarean and 21 vaginally. Table 2 lists the characteristics of women interviewed. In general, the women in Hangzhou had higher socio-economic status, in terms of education and employment, than those in Jiangshan, reflecting urban-rural differences. In addition, there was a substantial proportion of women older than 35; this reflects the unusual timeframe in which the study was conducted, in which older women were giving birth to second children immediately after the relaxation of the one-child policy. We note, however, that there has also been a gradual increase in births to older women over time given changes in family planning policy (see Table 1). In this paper, interviewees are referred to by reference initials: H for Hangzhou and J for Jiangshan; V for vaginal and C for cesarean; and F for first birth and S for second birth. We have also included their highest level of education attained.

**Table 2:** Sociodemographic characteristics of postpartum women interviewed

	Hangzhou	Jiangshan
Delivery Method		
Cesarean	13	11
Vaginal	11	10
Pain Relief Use/Support During Labor		
Epidural	3	1
Transcutaneous Electrical Nerve Stimulation (TENS)	0	8
Doula	3	0
Parity		
Primiparous	10	6
Multiparous	14	15
Mean age of first child ± SD (range)	7 ± 5.1 (2–21)	8.3 ± 5.2 (2–17)
Previous vaginal delivery	6	8
Previous cesarean delivery	8	7
VBAC	1	0
Age		
Mean ± SD (range)	32.5 ± 0.90 (25–44)	32.2 ± 1.28 (23–45)
20–24	0	2
25–29	6	6
30–34	10	6
35–39	7	5
40–45	1	2
Highest Educational Qualification		
Middle School	2	3
High School	1	9
Vocational college	1	6
Bachelor’s	17	3
Master’s and up	3	0
Currently Employed		
Yes	19	10
No	5	11

### Attitudes of primiparas

#### Vaginal birth as the primary choice

In both Hangzhou and Jiangshan, most low-risk primiparas stated that unless there were medical contraindications, they preferred vaginal births. The general remark for their stated preference was that vaginal birth was “better both for the baby and mother.” Some mentioned the benefits for the baby, including “fewer respiratory problems,” “better immunity” and “stimulation by squeezing through the vaginal canal.” However, more women emphasized the disadvantages of surgery: that recovery is slower and more painful, and surgery is harmful to the mother’s body. In addition, some explicitly mentioned the effects a cesarean delivery would have on future reproduction, including the spacing of future births and risk for further pregnancies:

“With vaginal birth, you only have to wait one or two years before having another. But if you had a C-section, you have to wait at the very least three years. That’s what I heard from my friend… Also, [with a previous C-section], you have an incision. What if you’re older? What if your first was small, and your second is bigger? Your belly is going to be stretched out even larger. It might rupture. So then there’s more risk, dangerous both to the mother and child.” (JVF3, vocational high school).

“The doctor will say, if you want to have a second child, you have wait at least five or six years. If you delivered vaginally, you can have another in one to two years, because you recover more quickly if you deliver vaginally. It’s better for the woman’s body and physique.” (HVF1, master’s).

One obstetrician at Jiangshan remarked that after the one-child policy was relaxed,

“It seems that more women at the hospital want natural birth because with a uterine scar, the second child is usually a cesarean birth. [A previous C-section] restricts the choice of delivery method and also adds some risks.”

#### Social norms and hospital promotion

Still, when asked why they decided to try for vaginal birth instead of elective cesarean delivery, most primiparas drew upon the fact that it was commonsense that women would do so. Their responses indicated that their decision was influenced by social norms—that it was what everyone else was saying and doing:

“They say that recovery is faster, and it’s better for the baby. So I wanted a vaginal delivery.” (JCF1, high school).

“With a cesarean, recovery is slower, and it might have more complications than vaginal birth. Usually women will all choose to have a vaginal birth.” (HVF4, vocational college).

“Everyone, at first, wants a vaginal birth. It’s only if something happens during labor that you get a C-section. Like my sister, when they told her she needed a C-section [before labor], she ran to the bathroom and cried…she wasn’t mentally prepared for a cesarean surgery—the news came so suddenly.” (JVF3, vocational high school).

Most received their information about childbirth from friends, family, the internet, and most importantly, the doctor or hospital. A 35-year-old Hangzhou primipara attributed the push for vaginal birth to widespread health promotion in hospitals, whether through doctor recommendations, pamphlets or prenatal classes:

“They’ve [the hospital] done health promotion pretty well. They really push natural birth. They constantly recommend natural birth, especially for the first child.” (HCF1, bachelor’s).

#### Role of the health care providers

While most women were motivated to try for vaginal delivery, many still worried about their mental and physical ability to give birth. Three primiparas, all of whom Jiangshan women, said they had thought about getting a C-section during labor because of the pain, but the nurse-midwives convinced them that they could persist with it:

“I did consider a cesarean birth [during labor] because it hurt so much. But the doctor said the baby was very small. They encouraged me to deliver vaginally, because there are more benefits to vaginal birth. If you are already hurting to that point, and you had the C-section, would you not suffer twice?” (JVF1, high school).

“During labor, I could not handle it. I was anxious, and I was afraid. I just wanted to get the baby out. When I requested a C-section, [the nurse-midwife] said ‘right now, there’s no problem so you should keep on going.’ She said that a C-section is not good for your recovery.” (JVF2, middle school).

On the other hand, only one college-educated primipara in Hangzhou specifically wanted and requested an elective cesarean delivery before labor, but was told she was not allowed to get it. She commented:

“I actually wanted a cesarean, because I feel like it isn’t as difficult or painful as vaginal birth. But, now if you are suitable for vaginal birth, you can’t get a cesarean section.” (HVF5, bachelor’s).

### Attitudes of multiparas with prior vaginal delivery

Many multiparas with prior vaginal delivery also preferred and tried for vaginal delivery for their second child, giving many of the same reasons as primiparas, with the added comment that there was no point to getting a surgery—and harming the body—if the first child was born vaginally. Only a few expressed a desire for a cesarean delivery pre-labor, either because they were afraid of the pain after having experienced it once, or because they were older and had been waiting for the change in policy to give birth to their second child. Still, like with the primiparas, doctors encouraged vaginal birth, telling them that labor was usually faster the second time and that CDs were not performed at the hospital without a medical indication. For example, one 44-year-old multipara with a previous vaginal birth wanted a CD:

“The problem is that for those who are older, everything has hardened. We don’t have any elasticity down there, so we can’t give birth. I fear that the child would get stuck and suffocate. My physique is not good—I’m too fat, too old.” (HVS4, middle school).

Another Jiangshan multiparas had previously delivered vaginally, but was overdue and anxious about the labor pain. She said that when she asked the doctor for a CD:

“The doctor recommended I give birth myself and give it a try. She also said that the second child might be faster and that it would be a pity if I had a C-section for the second child.” (JVS3, vocational college).

### Attitudes of multiparas with prior cesarean delivery

#### Previous culture of cesarean births

These pro-vaginal birth attitudes stand in sharp contrast to previously high rates of CD and previous birth experiences of multiparas who gave birth 5–10 years ago. According to both practitioners and these women, CDs were very common and widely accepted. As one Hangzhou nurse-midwife of 16 years put it,

“[The cesarean rate] was higher before because of ‘social factors.’ Women would get a cesarean just because their family members wanted one, they feared the pain, or for the slightest problem, like if the amniotic fluid was a bit low. Even women who had severe myopia would get cesareans. Now all these will try for vaginal birth.”

This culture resulted from practitioners’ encouragement of CDs and willingness to perform it for any reason, without consideration for medical indications, as well as women’s apparent low tolerance for risk and labor pain. CDs were perceived as a way to bypass the experience of labor. One 39-year-old college-educated multipara commented on the previous boom in CDs:

“People are finicky. We all knew that labor pain was very painful, and a lot of people did not want to bear it…And during that time everyone was only having one child, so they figured, they would just do the C-section and it would be fine. It also had to do with how people could bear hardship. Before [the widespread availability of CDs] there was no other way—you had to give birth yourself. Now, with this choice, of course people hoped that birth would go more smoothly.” (JCS7, bachelor’s).

The preference for CD was often justified by the one-child policy, since many women believed they would give birth just once. One multipara regretted requesting a cesarean nine years ago. She said recovering from her second cesarean surgery was much harder, and more painful, compared to the first:

“I thought I would only have one child! And I thought I might as well just get it over with with a cesarean...” (JCS1, bachelor’s).

Her husband added,

“The first time around I was worried she could not stand the pain. At the time, we were not allowed a second child so we never considered the risks. Right now, we regret that decision to get a C-section.”

Another woman in Jiangshan who requested a CD in 2013 said,

“During that time, I did not even consider [vaginal birth] an option because my belly was so big, and I did not think I would have another baby. [If I had known I would be able to have a second child,] I would have considered vaginal birth, because I heard that then having the second child is easier, and not as painful.” (JCS3, high school).

Doctors also admitted that CD indications are much stricter now. Strong messages discouraging CD and extolling the benefits of vaginal birth are consistently promoted through physician recommendations and prenatal classes. This has been reinforced by the relaxation of the two-child policy. Physicians have also become more pressured to manage reproductive complications related to previous CDs. One Hangzhou obstetrician remarked on the need to restrict unnecessary cesareans:

“Now we’ve realized that we did too many unnecessary cesarean deliveries. As OB/GYNs, we can see that these cesareans have consequences now. There are a lot of women who have cesarean scar ectopic pregnancies or placenta previa. Before there were not that many. There’s definitely a relationship there.”

#### Vaginal birth after cesarean (VBAC) as the safer option

While vaginal birth is the primary choice of primiparas or multiparas who have delivered vaginally, CD is the default delivery method for multiparas with previous CD. The Jiangshan hospital did not allow VBACs due to the lack of resources to handle possible uterine rupture, and accordingly, many women at Jiangshan did not know about the possibility of VBAC. Even if they did, they stated that no one else they knew attempted VBAC, and that risks of uterine rupture were high. Only one woman who adamantly wanted a VBAC was pressured by doctors not to:

“The doctors told my family that the rate of success [of a VBAC] is only 25%! They want to scare you. So then my family members said: ‘oh, C-section, C-section, let’s just do that.’” (JCS8, high school).

The Jiangshan physicians admitted that even though the risk of uterine rupture was comparatively low at 1%, they did not have the resources to monitor whether the uterus is ruptured, or respond appropriately, given that the operating room was on another floor of the hospital. One doctor said:

“You have to look at the conditions of the hospital. You have to ensure the patients’ safety. Only once this prerequisite is fulfilled, and only if they want to try vaginal birth, then we will let them try it. But how can we control these risks?”

She further said she did not feel comfortable with VBAC, because she had not handled many cases:

“A lot of it depends on the doctors’ skills. You have to assess all of the woman’s conditions, plus the details of the previous C-section. To tell you the truth, [the circumstances of the previous CD are] not easy to understand, because it might have been five to six years ago, or maybe it wasn’t done at our hospital. So I haven’t attempted to handle many VBACs.”

On the other hand, the Hangzhou hospital did allow VBACs under certain circumstances. Multiparas with previous CDs in Hangzhou were more aware of VBAC, largely from the internet, although some heard of it from their prenatal doctors. Many expressed hope to deliver by vaginal birth. However, almost all had a repeat CD; after doctors’ assessment of the thickness of the uterine scar and fetal size, they would be deterred by the risk or their likelihood of “success.” A multipara said,

“Originally I also wanted to try for vaginal birth. But the doctor did an assessment and saw [my scar] was too thin, like 0.7 millimeters thick. She said that a C-section was safer.” (HCS5, bachelor’s).

One 39-year-old multipara did not consider it at all:

“I had a uterine scar, which is risky. Also, I felt like I did not have any self-confidence to deliver vaginally. Add to that my age, I felt like the possibility of me giving birth vaginally was practically zero.” (HCS6, bachelor’s).

Only one woman interviewed gave birth by VBAC because she felt like a cesarean surgery was too painful, despite doctors’ comments that they themselves would choose a repeat C-section. But she had made her decision carefully:

“When they examined my scar, they saw that it was rather thick. I also had gotten pregnant two years after my first which is when the scar’s elasticity is the best. I did my own assessment and figured I could possibly deliver vaginally. If I had had any doubt I probably would not have considered it, because after all, safety comes first.” (HVS5, bachelor’s).

Doctors in Hangzhou were open to VBAC but were relatively conservative about it. Although they acknowledged that they had 24/7 anesthesia services, emergency surgery resources and on-site pediatrics teams to respond to uterine rupture, they believed cesarean birth was a much safer option. Therefore, though they offered VBAC to eligible women, they often recommended repeat CDs. One nurse-midwife in Hangzhou spoke of a patient who insisted on a VBAC, but had an emergency CD anyway:

“When I heard how tall she was, I felt like she would not be able to do it. She was only 1.5 meters. Her first was a C-section, [the baby was] around 3.5 kg. I did not recommend [VBAC], but her family members wanted her to try…but she ended up with a CD.”

One doctor in Hangzhou said that given the difficulty of labor and the added risk of uterine rupture, she could not face the responsibility of an adverse event occurring during VBAC:

“In China, the doctor-patient relationship is rather tense. If the patient does not have the desire to, we won’t force them to a vaginal birth…after all, there’s certain risks.”

### Use of and attitudes towards pain relief

Given that in previous literature fear of pain has been a significant factor in women’s decision to have a cesarean, we also inquired about their views of social support and pain relief during labor. In particular, we wanted to understand what role pain relief plays in women’s birth experience and how it affects or is tied to their perceptions of vaginal versus cesarean delivery. These experiences differed by hospital. In Hangzhou, most women knew about epidurals prior to labor. Of the 11 interviewees, three chose an epidural, while five felt like they could handle the pain without one. Three requested one, but were refused, mainly because certain standards were not met—either their cervix dilated too quickly or they were multiparous and doctors told them labor would be short. One primipara said:

“I adamantly wanted an epidural. But then my cervix dilated too quickly, so I could not use it in time… There are not many anesthesiologists here, and many women request epidurals. Even when you call for one, the time it takes the doctor to get there can’t match the speed of your cervix dilation. I was already sent to the delivery room before the anaesthesiologist got to me. I could only brace myself to give birth without it.” (HVF5, bachelor’s).

Still, when asked about their views towards epidurals, most women—even those that requested it—indicated that they were not too familiar with how it worked, that it might be ineffective, that it could have side effects, or that it would hurt to insert the epidural needle. As two women who opted not to use it remarked:

“I have heard of epidurals, but some people say it’s effective, others say it only slightly relieves the pain, and some say it doesn’t work, because everyone’s physique and ability to handle labor is different.” (HVS1, bachelor’s).

“I feared [the epidural] would be painful. They said they would insert the needle into the spine, and when I heard that, I thought: forget about it. In any case, I’ll go with labor pain—and there’s medical consequences to [the epidural]. I hear that people’s backs will hurt.” (HVS2, bachelor’s).

Of the three who did get an epidural, two remarked that the pain medication could only be used for a certain period before it ran out, and that it was only a temporary measure of relief:

“At 3cm they gave me an epidural. The epidural only works for two hours. Then they would add some more for a total of 3.5 hours. During that 3.5 hours, I had only opened from 3cm to 4cm. It wasn’t really effective. 4cm until 10cm, I had to depend on myself.” (HVF1, master’s).

On the other hand, more women were favorable and open to doula services, although this was also dependent on the availability of doulas.

“To be honest, I think that having someone with you [during labor] is more effective than [the epidural]. You’ll feel more calm, especially for your first child because you don’t know what it’s like.” (HVS1, bachelor’s).

“This time I did not ask for a doula, so I just lay on the bed. At that time, the doctor had inserted a drip. When it was all used up, I called for the doctor, but no one came over. Because no family members are allowed in, if there’s a person next to you, you’ll feel a little better. I think that doulas are great, if you have the money to get one.” (HVS2, bachelor’s).

In Jiangshan, women did not know about or decide to use any form of pain relief until nurses offered it in the labor-delivery room. Eight opted for transcutaneous electric nerve stimulation (TENS) which involved sticking pads on traditional Chinese medicine acupoints on the back and hand. TENS was the default form of pain relief offered, because it could be easily applied by the nurse-midwives. These women believed TENS worked, but only in the sense that it made labor shorter, not that it reduced pain.

“I used the stick-on pads, on the hands and back (TENS). The 1000 RMB one. They say it helps open the cervix. But as for the pain…it was still painful.” (JVS2, vocational college).

“Well, the nurses just asked me if I wanted [TENS], and I said I did. She explained to me the benefits, that I would give birth more quickly. I had already labored for a long time, so I decided to use it. I’m not too sure [if it was effective]. They say it is. I think that I gave birth more quickly. Labor was shorter.” (JVS5, middle school).

On the other hand, epidurals were available but not consistently offered in the labor-delivery room; as a result, not many women knew it was an option, and only one interviewee used it. When asked if she was offered an epidural, one primipara said,

“For vaginal birth? They don’t have that for vaginal birth, right? It seems like in the delivery room everyone used the electrode pads (TENS).” (JCF3, high school).

Of those who did know about or were offered epidurals, they, like the Hangzhou women, seemed to have a negative impression of it, or heavily depended on their doctors’ recommendation and guidance about pain relief options.

“[The nurses] don’t really promote the epidural, just the pads…They say that if you do the shot in the back, it will harm the back. If you stick the pads on, it’s like massage, it will be like electric stimulation. It was like a massaging sensation...But usually whatever the doctor says and we’ll listen.” (JVS1, college).

“The stick-on ones don’t have any side effects. But if you have an injection, then it might affect your body. This is my impression. At the very least you have a needle stuck into you.” (JVF3, vocational high school).

“If they did a shot, then, that might as well be a C-section—that’s anesthesia. They only give anesthesia with a C-section. In any case, no matter C-section or vaginal birth, it’s all painful.” (JVF2, middle school).

“I was afraid there could be side effects [to the epidural]. I did not trust it. My family is very traditional. They definitely won’t agree to using *wutong*.” (JVS4, vocational college).

Physicians and nurse-midwives looked favorably upon the introduction of pain relief methods within the past few years, although they viewed it as the woman’s choice, except when contraindicated. One nurse-midwife at the Hangzhou hospital said:

“With an epidural, it depends on whether they know about it or their ability to accept it. Some people feel like epidurals are great because it can relieve the pain. If they fear the pain, of course they hope to use it. Some think that because it’s a drug, they don’t want to use it. Most people who have understood that it’s a widely-recognized, safe method would accept it.”

However, non-epidural methods were more widely accepted and promoted, because of the epidural-related issues of both anesthesiologist availability and potential side effects, such as its association with lengthening labor and increasing risk of instrumental delivery.

“We promote natural birth… there are also other [non-pharmacological] methods of pain relief to ease the pain [referring to also doulas, massage, water showers, and Lamaze breathing]. The end goal is that they can give birth vaginally.” (Hangzhou Nurse-Midwife).

“[The use of epidurals] depends on the doctors’ assessment. We also have to see if we can do it—like if the anesthesiologist is very busy, if it’s nighttime, or if they’re in surgery, then we won’t do the epidural. Most people will choose [TENS] because you just need a nurse to put it on and that’s it. But for some patients, if they did not sleep the entire night, we’ll do an epidural for them so they can rest.” (Jiangshan OB/GYN).

## Discussion

In 2008, China was purported to have one of the highest rates of cesarean delivery in the world [2]. In the span of just 10 years, rates in urban centers decreased substantially, and the rate of increase in other areas slowed [16]. Such rapid change is unprecedented. The new culture is in sharp contrast to previous encouragement by doctors to undergo CD, and the frequency of maternal requests [5, 22, 23]. In addition, in 2016 China officially ended the one-child policy, creating significant implications for maternity care. This study is the first to examine delivery decision-making at the intersection of these two phenomena. Given the unusual period in which this research was conducted, our interviewees included a disproportionate number of older women with previous CDs. While this unusual situation may not persist, the cohort interviewed does give us some insight into how the culture of delivery decision-making has changed over time.

From our interviews, we found a strong preference and willingness to try for vaginal births among primiparas in both urban and rural settings, representing a huge shift from previous years. Past studies showed that up to 25% of CDs were CDMR, often requested by urban, educated women [5, 9, 22, 24]. In this study, no woman purportedly had CDMR, and in fact, most women of higher socioeconomic status supported vaginal birth. Given that the proportion of CDs had started to drop before changes to the family planning policy were announced, we believe that government policy and promotion have influenced physician suggestions affecting women’s choices, as well as women’s beliefs about delivery. The relaxation of the one-child policy has further reinforced this change in mindset. The two-child policy has increased awareness of the risk of CD for future pregnancies [25–28]. Previously the impossibility of future births gave women the impetus to request CD, a decision some came to regret for their second pregnancy [1, 5, 29]. This highlights the unintended consequences China’s family planning policy has had on delivery decision-making.

We found that regardless of setting, hospitals and practitioners have been key to driving change in delivery method preferences and decision-making. CDs had been encouraged by doctors for convenience, financial and defensive reasons [15, 30, 31]. Moreover, past studies have shown that physicians themselves preferred CD, which can influence maternal preference [29, 31, 32]. However, this is changing. Physicians, at least in Jiangshan and Hangzhou, have become more adherent to medical indications for CD, and they themselves now promote vaginal delivery in most circumstances. They also no longer consider CD as an elective choice for low-risk women, which may also raise questions of maternal autonomy in delivery decision-making.

While the norms for low-risk primiparous women are similar across rural and urban settings, differences in childbirth knowledge, attitudes and experience emerged specifically in two areas: repeat CD and pain relief during labor, which often stemmed from variations in availability of resources and information. In China, repeat CD is the default delivery method for women with previous CD, which suggests the decrease in CD rates has been mainly driven by prevention of the primary CD. Potential risks of uterine rupture mean that VBACs, like in other countries, are not offered, as in Jiangshan, or are advised against, as in Hangzhou [33–37]. The availability of VBAC and the childbirth environment thus shaped women’s perceptions about their childbirth options after CD. As more women are having second children, Chinese hospitals will need to carefully consider the integration of VBAC as a viable delivery method for women with previous CD. As in other contexts, particular attention will have to be paid to childbirth environment, management of labor and perceptions of risk [38–41].

While interviewees’ responses demonstrate how delivery preference and decision-making have changed, we also observe that the environment of birth has become more patient-centered, particularly at tertiary-level hospitals. Previous studies have shown how lack of pain relief and social support can drive requests for non-medically-indicated CD [20, 42, 43]. While women did not explicitly connect their birth environment to their decision for vaginal birth, they are becoming aware of and choosing options to ease labor. In Hangzhou, and to a lesser extent Jiangshan, hospitals are promoting freedom of movement during labor, limiting interventions such as episiotomies, and offering private labor and delivery rooms (although at a higher cost)—all options that promote vaginal over cesarean birth. Still, women continue to express anxiety about birth, and certain barriers continue to exist particularly at secondary-level, more rural hospitals: most women still labor alone in open wards, and volume of care continues to be high with limited availability of staff [15, 42, 44]. Moreover, while awareness of pain relief is spreading, the majority of women, particularly in Jiangshan, continue to express negative views of or mistrust towards pharmacological or anesthetic forms of pain relief. This may be because pain relief options are limited, and services are not necessarily guaranteed or widely promoted. Women’s responses also indicate that social support and one-on-one encouragement may be seen as a better option to relieve the suffering of labor; however, in China, this is also constrained by the scarcity of resources, particularly at public hospitals [20, 42]. While women’s delivery preferences may be influenced by doctors’ suggestions or government promotion, they are not enough to ensure the most comfortable childbirth experience or environment.

In a context where many other countries’ CD rates are increasing, China provides an example of how top-down efforts can not only reduce CD rates, but also change the perception of childbirth and culture of delivery decision-making. After the release of a 2007–2008 WHO report indicating high rates of CD in China and associating unnecessary CDs with higher rates of maternal mortality and morbidity, the Ministry of Health and the Chinese Maternal-Child Health Association launched a campaign to “Promote Natural Birth and Ensure Maternal-Child Health” [2, 45, 46]. This campaign consisted of various efforts: assessing and adjusting hospital standards, training physicians to follow a less-interventionist approach to childbirth, and disseminating material promoting natural birth [47, 48]. In addition, CD rates became an indicator of hospital quality, providing hospitals’ an impetus to reduce CD rates. Provincial governments began offering financial incentives or disincentives to hospitals to decrease their CD rates, which, in turn, filtered down to the doctors to reduce CDs performed [49]. As can be seen from our interviews, such efforts have had an enormous effect on delivery decision-making, maternal preference, and physician behavior. This success emphasizes the social, cultural and economic nature of CD rates, and how they can be addressed. They also provide a lesson for many other countries with high CD rates.

This study has a few limitations. First, the sample was non-randomly drawn from only two hospitals in Zhejiang Province, and is clearly not generalizable to the rest of China, particularly less-developed areas. However, evidence shows that CD rates are decreasing in many cities where they had previously reached 50–70%, so the situation described in this study is probably mirrored elsewhere [16]. Another limitation was that our interviews focused on the woman and healthcare provider; in China, the husband and the family are also important participants in the decision-making process. Selection bias and response bias could have also been a factor; because interviews were conducted in a hospital setting, interviewees may have been more likely to respond according to doctors’ wishes. However, given the researcher was not affiliated with the hospital, they seemed to talk candidly about their experiences. Finally, we only interviewed postpartum women, whose responses might have been affected by ex-post rationalization; further research should follow and interview women throughout their pregnancy and after delivery to better describe the process of childbirth decision-making.

## Conclusion

In China, changes in family planning policy and efforts to promote vaginal birth have greatly changed the culture of delivery decision-making, leading to decreased CD rates. This demonstrates the powerful role social factors and public policy can play in CD rates, and might provide suggestions for other countries wishing to lower CD rates. At the same time, it also exemplifies the unintended effects of family planning policies on delivery decision-making. Research should further examine changes in CD and other reproductive decisions during this new multiparous era.

## Additional files

Additional file 1: Interview Guide for Mothers. A semi-structured interview guide for mothers participating in the study. (DOCX 18 kb)

Additional file 2; Interview Guide for Doctors-Nurses. A semi-structured interview guide for providers participating in the study. (DOCX 17 kb)

## Abbreviations

CD: Cesarean delivery
OB/GYN: Obstetrician-gynecologist
TENS: Transcutaneous electrical nerve stimulation
VBAC: Vaginal birth after cesarean
