# Supplementary material for: Large reductions in cesarean delivery rates in China: a qualitative study on delivery decision-making in the era of the two-child policy
Source: BMC Pregnancy Childbirth. 2017 Dec 4;17:405. doi: 10.1186/s12884-017-1597-9 (PMC5716234; doi:10.1186/s12884-017-1597-9)
Supplement: Supplementary file 1 — Interview Guide for Mothers. A semi-structured interview guide for mothers participating in the study. (DOCX 18 kb) [file 12884_2017_1597_MOESM1_ESM.docx]

**Interview Guide with Postpartum Mothers**

**DEMOGRAPHICS**

- How old are you?
- What is your level of education?
- What do you do as part of your work?
- What type of insurance do you have? How much did you pay for your care so far?
- Where are you from?

**CHILDBIRTH EXPERIENCE**

- How was your childbirth experience? Please describe your pregnancy, labor and birth.
- Did you conceive by IVF or other means of fertility treatment?
- Influence of family members/doctors:
  - What did your doctor tell you about your baby?
  - Did your prenatal doctor or OB suggest that you have a (VD or CD)? Or did you think they had a preference for your mode of delivery?
  - If they told you needed a C-section, did they tell you why?
- What did you know about childbirth before your delivery?
  - Did you take a child birthing course or been educated on labor?
  - What prior knowledge of other women’s experiences of birth did you have?
    - Did your mother tell you anything? Friends?
- Were you afraid of giving birth or anxious about it? What scared you most?
  - Did you fear the pain? Did you feel like you could cope with it?
- What do you think of cesarean versus vaginal delivery?
  - Which one is more painful?
  - Which one is more safe for you or for your baby?
  - [If elective C-section]: Why did you choose to have a C-section?
- Did you have the option of pain relief? Did you choose to use it?
  - If so,
    - What did you have?
    - Did it work?
  - If not, why not?

**PREVIOUS/FUTURE CHILDREN**

- Was this your first child?

If YES:

- - How did you give birth? Describe your birth experience.
  - Did you want to give birth this way? Why?
  - Do you plan on having another child? If so, does this influence this choice to have a [vaginal/cesarean] delivery? If not, why not?
  - Did you have a preferred gender for this child?

If NO:

- - How many children did you have previously?
  - Where and when did you give birth to your previous children?
  - How did you give birth (vaginal, cesarean, emergency cesarean)? Describe your birth experience.
    - Was that your preferred method of birth?
  - **When, how and why did you decide to have another child?**
    - Influence of family members?
    - [If older, 35-40 years old] did you hesitate to have a child at an older age?
      - Were you worried about being able to conceive?
    - How will this affect your career and/or family life?
  - How did you plan to give birth to this child?
    - [VBAC, Cesarean, ask about doctors’ expertise]
  - If you had known you were to give birth again, would that have changed your previous preferred method of delivery?
    - If you had a C-section for uterine scar, did you want to have a second CS or elect to have a VBAC? Why or why not?
      - Which do you think is riskier for you? For the child?
    - Do you have a preferred gender for this child?

**TWO-CHILD POLICY, TAKING CARE OF CHILDREN**

- What do you think of the two-child policy and how has that affected family planning for either you or your friends?
  - Do you know of any women your age who decided *not* to have second children? Why?
- Who takes care of the children/ will take care of the children? What are your plans for raising your children? Hopes and dreams?
