# Supplementary material for: Large reductions in cesarean delivery rates in China: a qualitative study on delivery decision-making in the era of the two-child policy
Source: BMC Pregnancy Childbirth. 2017 Dec 4;17:405. doi: 10.1186/s12884-017-1597-9 (PMC5716234; doi:10.1186/s12884-017-1597-9)
Supplement: Supplementary file 2 — Interview Guide for Doctors-Nurses. A semi-structured interview guide for providers participating in the study. (DOCX 17 kb) [file 12884_2017_1597_MOESM2_ESM.docx]

**Interview Guide for Doctors and Nurses**

- What is your name?
- What is your educational experience? Training experience?
  - Why did you decided to become an OB/GYN?
- How long have you worked as a doctor/nurse in this hospital?

**TWO-CHILD POLICY AND ITS EFFECTS**

- What are the trends do you see in women giving birth since 2016 (in the past decades)?
  - Are there more women giving birth?
  - Are they older?
  - *Who* tends to have a second child?
- How has the overall cesarean section rate changed over time, especially with the new policy change?
- **VBAC/ERCS**
  - For women who previous had C-sections, do you recommend VBAC or ERCS?
    - Are you performing more cesarean deliveries for women who are having second children?
    - Have there been any complications of this?
    - Do you have the training for handling complications for a vaginal birth after cesarean delivery?
    - Have you seen women who had a strong preference for either VBAC or ERCS?
  - Are you worried about the risk of cesarean deliveries in subsequent pregnancies?
  - How do you counsel primiparous women who want to have a cesarean section?
- Do women often ask you do a cesarean section for them? How often?
  - What is the main reason they ask for a cesarean?
  - If a mother asks you for a cesarean section for her child, what do you say or do? Will you do it for them?
  - Do families of the mother ever request for a cesarean? Do you interact with the family at all?
- What is the cost of vaginal birth or cesarean birth at this hospital?

**PAIN RELIEF DURING LABOR**

- Are epidurals or labor analgesia available at this hospital?
  - Are women aware of pain relief options?
  - What do you think about pain relief methods during labor?
    - Do you support giving epidurals to women?
  - Why are anesthesiologists willing to administer anesthesia for cesarean sections but not for laboring women?
  - What is the cost of pain relief?

**FERTILITY, MISCARRIAGES AND IVF**

- As an OB/GYN, have you seen an increase in the number of women who trying to conceive? Have there been incidences of miscarriages?
- What about conception by IVF and other fertility methods? Who tends to get these?
  - If they conceive by IVF, do you recommend vaginal or cesarean birth?

**PERSONAL**

- [If Female] Have you given birth before? What was your experience?
  - What mode of delivery did you prefer for yourself or (if male) your wife?
  - Why?
- [If Female] Have you wanted to have another child since the policy change? How has that affected your career?

**HEALTH SYSTEM**

- What is it like being an obstetrician here? Tell me about problems with the current system of health care in your experience.
  - What do you like most about your career? The least?
  - Do you think there is a pressure on you as a doctor to have good outcomes? Do you fear being sued or medical malpractice as an OB/GYN?
- What was the scariest/most memorable moment in your career as an OB/GYN?
- Do you have anything to add about this topic?
